# The effects of manual therapy in pain and safety of patients with knee osteoarthritis: a systematic review and meta-analysis

**DOI:** 10.1186/s13643-024-02467-7

**Published:** 2024-03-19

**Authors:** Bowen Zhu, He Ba, Lingjun Kong, Yangyang Fu, Jun Ren, Qingguang Zhu, Min Fang

**Affiliations:** 1 Shuguang Hospital, Shanghai University of Traditional Chinese Medicine, Shanghai, China; 2 Department of Integrative Oncology, Shanghai Cancer Center, Qingdao Institute, Fudan University, Qingdao, China; 3 Department of Traditional Chinese Massage, Yueyang Hospital of Integrated Traditional Chinese and Western Medicine, Shanghai University of Traditional Chinese Medicine, Shanghai, China; 4 Institute of Traditional Chinese Medicine and Massage, Shanghai Academy of Traditional Chinese Medicine, Shanghai, China

**Keywords:** Manual therapy, Knee osteoarthritis, Pain, Safety, Meta-analysis

## Abstract

**Background:**

Manual therapy (MT) is frequently used in combination with management of osteoarthritis of the knee, but there is no consensus on the exact efficacy of this treatment strategy. The purpose of this systematic review and meta-analysis was to evaluate the pain relief and safety of MT for treatment of knee osteoarthritis (KOA).

**Methods:**

Randomized controlled trials evaluating MT in patients with KOA in major English and Chinese journals were searched in the following databases: Wanfang, China Science and Technology Journal Database (VIP database), China National Knowledge Infrastructure (CNKI), PubMed, Embase, Web of Science, and the Cochrane Library databases through June 2023. The methodological quality and quality of evidence of the included studies were assessed using Cochrane’s risk-of-bias 2 (ROB 2) tool and the Grading of Recommendations Assessment, Development, and Evaluation (GRADE) tool. Data analysis was performed using Stata version 15.0 software. After use of Galbraith plots to exclude studies that could lead to heterogeneity, random effects models were used to analyze the remaining data and test the consistency of the findings. We used meta-regression to assess the effect of treatment period, patient age, and sex ratio on outcomes. Funnel plots and Egger’s test were used to evaluate publication bias. Sensitivity analyses were used to determine the reliability of the results.

**Results:**

A total of 25 studies, with 2376 participants, were included in this review. The overall methodological quality of the included studies was limited. Our findings suggest that MT has a positive impact on pain relief outcomes in KOA patients. The meta-analysis showed that MT was superior to usual care (*SMD* = 2.04, 95% *CI* 0.94, 3.14, *I* ^2^ = 96.3%; low evidence quality) and exercise (*SMD* = 1.56, 95% *CI* 0.41, 2.71, *I* ^2^ = 96.3%; low evidence quality) for reducing pain. In terms of improvement in visual analogue scale (VAS) scores, MT treatment beyond 4 weeks (*SMD* = 1.56, 95% *CI* 0.41, 2.71, *I* ^2^ = 96.3%) may be superior to treatments less than or equal to 4 weeks (*SMD* = 1.24, 95% *CI* 0.56, 1.95, *I* ^2^ = 94.7%). No serious adverse events associated with MT were reported.

**Conclusions:**

MT may be effective at reducing pain in patients with KOA and may be more effective after a 4-week treatment period. Compared with usual care and exercise therapy, MT may be superior at reducing KOA pain in the short term (9 weeks), but its long-term efficacy requires careful consideration of evidence-based outcomes. MT appears to be safe for KOA patients, though clinicians should inform patients of the potential risk of MT-related adverse events.

**Supplementary Information:**

The online version contains supplementary material available at 10.1186/s13643-024-02467-7.

## Background

Knee osteoarthritis (KOA) is a chronic degenerative disease of the knee joint, and knee joint pain is the most common clinical manifestation [1]. The worldwide prevalence of radiographically confirmed symptomatic KOA is estimated to be 3.8%, and the prevalence of this disease has increased to more than 10% in people older than 60 years [2]. In China, the prevalence of KOA among older people is approximately 8.5% [3], and the incidence of KOA has increased significantly among younger people [4]. KOA has a serious impact on the health status of patients and overall quality of life and can even cause a serious economic burden on society [5]. Clinical guidelines recommend that relieving pain in the knee is a primary target of KOA treatment [6, 7].

Manual therapy (MT), including massage therapy and manipulative therapy, is a widely used conservative treatment strategy [8]. MT may have a positive effect on reducing pain [9, 10] and is reportedly used by approximately 15.4 million people in the USA for treatment of KOA, for example [11]. In some countries, MT is considered a first-line treatment option [12], whereas in others, it is recommended to be used as part of a broader treatment program that includes exercise; alternatively, MT is not recommended because of a lack of evidence [13]. Despite widespread use of MT, few studies have reported the efficacy of MT alone for treatment of KOA, with poor methodological quality [14]. Due to the lack of evidence, there is no consensus about recommending MT for KOA patients.

In recent years, several randomized controlled clinical trials (RCTs) have been conducted to assess the efficacy of manipulation in management of KOA, particularly in China. Indeed, Chinese massage therapy, including soft tissue manipulation and joint manipulation, has been used by practitioners for management of KOA, and trials have shown good results in terms of reducing pain [15, 16]. Therefore, additional evidence-based evidence is needed to explore the analgesic efficacy of MT.

This study was conducted by performing a systematic review and meta-analysis to assess the benefits of MT alone in management of KOA, mainly in terms of improving pain and updating the effects of MT on patients with KOA, to provide empirical evidence and reference for clinical application of MT in treatment of KOA.

## Methods

The protocol was registered after all phases of the review process were completed, and the manuscript was finished. This study was performed strictly by following Preferred Reporting Items for Systematic Reviews and Meta-Analyses (PRISMA) statement guidelines [17]. The INPLASY registration number was INPLASY 202360030.

### Literature search

We conducted a literature search of the following databases for articles published from inception to December 2021 with the language restricted to English or Chinese, with an updated search conducted in July 2023: Wanfang Database, China Science and Technology Journal Database (VIP database), China National Knowledge Infrastructure (CNKI), PubMed, Embase, Web of Science, and the Cochrane Library databases. All keywords were mapped to “indexed items” (e.g., MeSH) using a combination of the following in Embase.

#1'knee'/exp.

#2knee*:ti,ab,kw.

#3[< 1966–2023]/py.

#4 (#1 OR #2) AND #3

#5'arthritis'/exp OR 'osteoarthritis'/exp.

#6arthrit*:ti,ab,kw OR osteoarthr*:ti,ab,kw.

#7[< 1966–2023]/py.

#8 (#5 OR #6) AND #7

#9'massage'/exp OR 'musculoskeletal manipulation'/exp.

#10massage*:ti,ab,kw OR 'zone therap*':ti,ab,kw OR manipul*:ti,ab,kw.

#11[< 1966–2023]/py.

#12 (#9 OR #10) AND #11

#13'western ontario and mcmaster universities osteoarthritis index'/exp OR 'visual analog scale'/exp.

#14'western ontario and mcmaster universities osteoarthritis index*' OR womac.

#15'visual analog scale' OR vas.

#16 #13 OR #14 OR #15

#17'randomized controlled trial'/exp OR 'randomized controlled trial (topic)'/exp OR 'controlled clinical trial'/exp OR 'randomization'/exp OR 'double blind procedure'/exp OR 'single blind procedure'/exp.

#18 (clinic* NEAR/2 trial*):ti,ab,kw.

#19random*:ti,ab,kw OR placebo*:ti,ab,kw OR blind*:ti,ab,kw OR mask*:ti,ab,kw.

#20 #17 OR (#18 AND #19)

#4 AND #8 AND #12 AND #16 AND #20

The detailed search strategy is shown as an example in Appendix 1.

### Study selection

Only RCTs that reported the method of randomization to MT alone for KOA were included. In the case of a three-arm or multiarm RCT, articles were included if two of the groups met the inclusion criteria [18]. All patients with KOA were included, regardless of age, race, sex, age limit, or severity. If a study did not report information on the randomization method, ethics approval, or clinical study registration, it was excluded. Case reports, empirical reports, and laboratory studies were not included.

### Eligibility criteria

Patients included in the study had a clear diagnosis of KOA and met other diagnostic criteria, such as the American College of Rheumatology criteria or the Chinese Medical Association Orthopaedic Branch Guidelines for the Treatment of Osteoarthritis (2018), with no restrictions on the severity of the disease. Interventions in the experimental group involved only MT, and those in the control group involved any therapy other than MT (such as acupuncture treatment, medication, exercise, and usual care). In addition, if the observed differences were thought to be due to the unique contribution of MT, we included studies that may encompass research in which MT was provided as part of a package of care, that is, if the effects of MT could be isolated. For example, studies comparing MT plus usual care with usual care alone were included, whereas investigations comparing MT plus usual care with MT alone were not. We also excluded studies in which MT was combined with other therapies because it was difficult to distinguish the effect of MT.

### Outcome analyses

The effect of MT in combination with any other therapeutic adjuncts (including usual care, herbal application, oral analgesics, exercise, acupuncture) was examined. The primary outcome was the VAS pain assessment scale [19]. Secondary outcomes were the Western Ontario and McMaster Universities Osteoarthritis Index (WOMAC) pain scale [20], follow-up data, and adverse events.

### Data extraction

Two authors (B. Z. and H. B.) independently collected the data, including the following: number of subjects, sex, age, body mass index (BMI), duration of disease, country, MT intervention period, diagnostic criteria for KOA, Kellgren–Lawrence grade, VAS score, WOMAC pain score, follow-up duration, and incidence of adverse events. In the meta-analysis, VAS and/or WOMAC pain scores were extracted. In the absence of sufficient data, the corresponding authors were contacted for additional data.

### Risk-of-bias assessment

Risk of bias in RCTs was assessed according to the revised Cochrane risk of bias in randomized trials tool (RoB2) [21]. All risk-of-bias assessments were conducted in duplicate by 2 reviewers (L. K. and R. J.). Disagreements were resolved by recruiting a third author (Y. F.) to reach a consensus.

### Quality of the evidence: Cochrane GRADE assessment

The Grading of Recommendations, Assessment, Development and Evaluation (GRADE) methodology [22] is a system for grading the quality of evidence for health recommendations and assessing the quality of evidence [23]. The quality of the evidence is rated from “very low” to “high”: (a) high (the true effect is considered close to the estimated effect), (b) moderate (confidence in the estimated effect is moderate), (c) low (confidence in the effect is limited), and (d) very low (confidence in the effect is very limited, and there is a high degree of uncertainty about the outcome) [24]. Scores were reviewed by the senior author (L. K.).

### Data synthesis and analysis

All the search results were imported into NoteExpress v3.5.0.9054 for management. Two reviewers independently screened all potentially eligible studies. Titles and abstracts were screened first to exclude irrelevant citations. The full texts of all articles with potentially relevant abstracts were retrieved and screened according to the study eligibility criteria. Disagreements were resolved by consensus or discussion with a third reviewer.

The primary endpoint of this meta-analysis was the difference in VAS score between patients receiving MT and those receiving other therapies. Adverse events are summarized narratively. All pain scales were converted to a 10-point scale. The secondary endpoint was the difference in WOMAC pain scores (0–20) between these two groups of patients. The negative effect size of the VAS score or WOMAC score indicates that MT was more beneficial than other therapies, indicating that the participants had less pain. Primary and secondary outcomes were collected, and the data were analyzed after randomization and at the end of treatment. Minimum clinically important difference (MCID) thresholds for the VAS and WOMAC scores were defined as a 20% fluctuation from the baseline of the included studies based on previous studies and were calculated as follows: 1.18/10 for the VAS for pain and 2.12/20 for the WOMAC pain score [25].

We extracted final value scores (means and standard deviations) for the meta-analysis and converted change scores into mean values [26, 27]. Heterogeneity among the included studies was assessed using the *Q*-test and is presented as *I* ^2^ and *P*-values. An *I* ^2^ > 50% and/or a *P*-value < 0.1 indicated significant heterogeneity among the studies. If the heterogeneity test showed significant heterogeneity, a random effects model was used; otherwise, a fixed effects model was applied [28]. To explore the source of heterogeneity, we conducted subgroup analysis of VAS scores based on differences in treatment methods. The subgroup analysis of VAS scores was based on the course of treatment (set cut-off value of 4 weeks); although we considered the effect of the course of MT because it was not included in our previously designed protocol, we included this comparison because it represents a point for continuing discussion. We also used the Galbraith plot to explore studies that may have contributed to the heterogeneity and excluded them.

Then, we used a random effects model to analyze the remaining data, and we observed that the results were consistent with those obtained previously [29]. Meta-regression was used to evaluate the effect of the treatment period and sex ratio on the results, and the threshold for statistical significance was set at *P* < 0.05. We used a funnel plot and Egger’s test to evaluate publication bias (*P* < 0.1 was considered to indicate significant publication bias among the enrolled studies). To assess the reliability of the findings in this study, sensitivity analyses were also conducted. All analyses were carried out with Stata version 15.0 (Stata Corporation, College Station, TX, USA).

There were no public or patient representatives directly involved in the drafting or process of this review.

## Results

### Search and selection

**Fig. 1:**
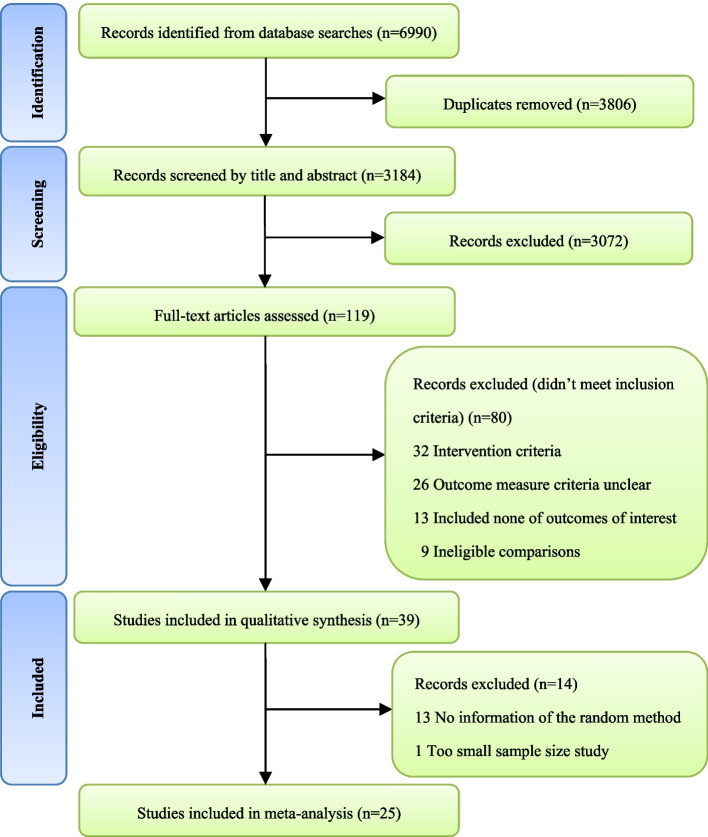
Selection of studies through review

The literature search identified 6990 records, and a total of 3184 potential studies were identified after removing duplicates. After screening the titles and abstracts, 3072 studies were excluded, and the remaining 119 full texts were screened for inclusion. After the detailed full-text screening, 39 studies were included in the qualitative synthesis. Finally, 25 RCTs were included in the current review [30–54]. The detailed process of the search and selection is shown in Fig. 1.

### Risk-of-bias assessment of pain outcomes

**Fig. 2:**
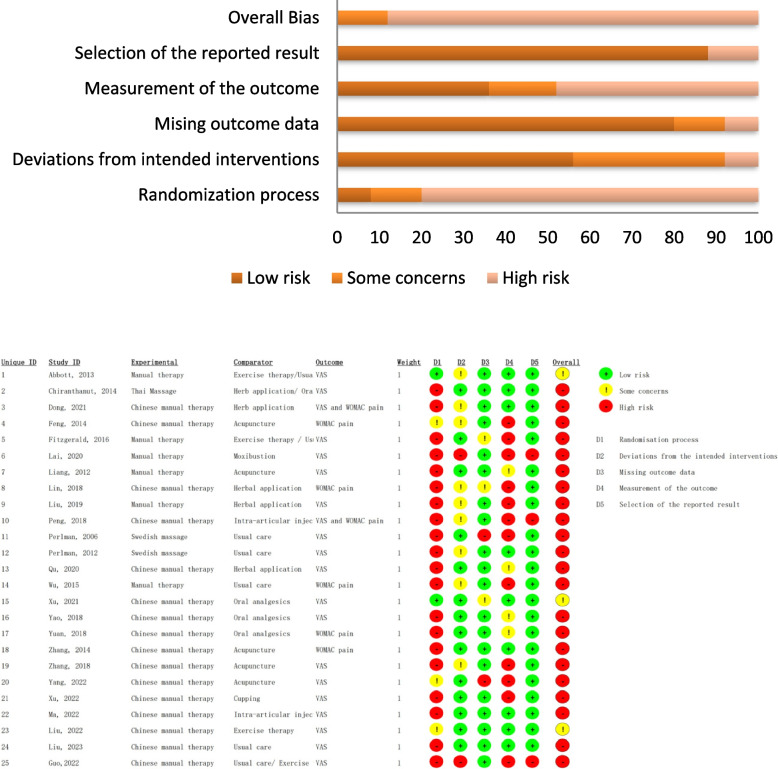
Results of the risk-of-bias assessment using RoB2

Three of the 25 included studies were considered to have a moderate risk of bias [22, 30, 44]; the remaining 22 studies all showed a high risk of bias. The randomization process revealed some concern, with a high risk of bias. The common areas of bias were selection bias (allocation concealment) and performance bias (blinding of participants and/or healthcare providers). All the included studies failed to meet at least one of the two criteria. The detailed risk-of-bias assessment of pain outcomes is shown in Fig. 2 and Appendix 2.

### Effects of interventions

The treatment effects, quality of the evidence, and GRADE summary of VAS scores for all comparisons among the included trials are summarized in Table 1. The GRADE approach to evidence synthesis and operationalization of criteria items are shown in Appendix 3.

**Table 1:** GRADE summary of VAS for all comparisons among trials included

Comparisons	Assessment time	Studies	Effect estimate* (95% CI)	Participants	*I* ^2^ (%)	Quality of evidence
**MT vs usual care**	At the end of the intervention	Abbott (2013) [30] Fitzgerald (2016) [34] Perlman (2006) [40] Perlman (2012) [41] Liu (2023) [53] Guo (2022) [54]	2.04 (0.94, 3.14)	500	96.30	**Low**
**MT vs exercise**	At the end of the intervention	Abbott (2013) [30] Fitzgerald (2016) [34] Liu (2022) [53] Guo (2022) [54]	1.56 (0.41, 2.71)	421	96.20	**Low**
**MT vs herb application**	At the end of the intervention	Chiranthanut (2014) [31] Dong (2021) [32] Liu (2019) [38] Qu (2020) [42]	0.04 (− 0.49, 0.57)	410	83.50	**Low**
**MT vs oral analgesics**	At the end of the intervention	Chiranthanut (2014) [31] Xu (2021) [44] Yao (2018) [45]	0.55 (− 0.08, 1.18)	197	77.50	**Very low**
**MT vs acupuncture**	At the end of the intervention	Liang (2012) [36] Zhang (2018) [48] Yang (2022) [49]	− 0.53 (− 1.38, 0.33)	568	92.20	**Low**
**MT vs intra-articular injection**	At the end of the intervention	Peng (2018) [39] Ma (2022) [51]	0.47 (− 0.91, 1.86)	172	94.20	**Very low**
**MT vs moxibustion**	At the end of the intervention	Lai (2020) [35]	N/A	40	N/A	**Very low**
**MT vs cupping**	At the end of the intervention	Xu (2022) [50]	N/A	68	N/A	**Very low**

*MT* manual therapy, *N/A* not applicable

### Outcomes

#### MT compared with other therapies in VAS score of KOA patients

### The included studies and population

Twenty trials (25 cohorts) published between 2013 and 2023 included 2376 patients (mean age was 61.38 years) and reported changes in the VAS scores of KOA patients who received MT or other therapies. Eleven trials [31, 32, 36, 38, 44, 45, 49–53] reported the severity of the disease in patients using the Kellgren-Lawrence criteria, and the remaining studies included patients with severity levels of III or less, except for two trials [31, 38] that were graded IV. Five trials were published in English and 15 in Chinese. A detailed description of the characteristics of the studies is available upon request from the primary author. The sample sizes of the studies ranged from 40 to 448. The detailed characteristics of each included study are listed in Table 2.

**Table 2:** Characteristics of included studies and patients

Author (year)	Country	Number of centers	Total number of randomized participants	Gender (male/female)	Age, mean ± SD (years)	Duration of KOA	K/L grading	Intervention	Control condition	Outcome measure	Adverse events
Abbott (2013) [30]	New Zealand	1	156	71/85	67.30 ± 10.20 E 66.90 ± 8.20 C 66.10 ± 10.70 C	2.50 ± 1.40 E, Y 2.60 ± 1.40 C, Y 2.80 ± 1.30 C, Y	NR	Manual therapy	Exercise therapy/usual care	VAS	-
Chiranthanut (2014) [31]	Thailand	1	60	16/44	65.45 ± 9.75 E 63.70 ± 6.07 C 62.25 ± 9.50 C	7.37 ± 7.16 E, Y 6.45 ± 4.87 C, Y 7.95 ± 8.20 C, Y	II −IV	Thai massage	Herb application/oral analgesics	VAS	+
Dong (2021) [32]	China	1	210	138/72	61.90 ± 6.70 E 62.40 ± 7.30 C	2.40 ± 0.70 E, Y 2.90 ± 0.30 C, Y	0 − II	Chinese massage	Herb application	VAS and WOMAC pain	-
Feng (2014) [33]	China	1	62	21/41	62.13 ± 4.47	2.89 ± 0.31, Y	NR	Chinese massage	Acupuncture	WOMAC pain	NR
Fitzgerald (2016) [34]	USA	3	226	74/152	58.30 ± 9.80 E 58.40 ± 8.70 C 58.00 ± 10.00 C	< 1 Y 26 1–2 Y 29 3–5 Y 46 5–10 Y 70 > 10 Y 55	NR	Manual therapy	Exercise therapy/usual care	VAS	+
Lai (2020) [35]	China	1	40	17/23	65.29 ± 10.55 E 65.70 ± 10.16 C	4.12 ± 0.50 E, Y 4.03 ± 0.51 C, Y	NR	Manual therapy	Moxibustion	VAS	NR
Liang (2012) [36]	China	1	60	16/44	56.17 ± 9.48 E 58.26 ± 11.92 C	13.24 ± 6.53 E, M 10.75 ± 7.12 C, M	0–II	Manual therapy	Acupuncture	VAS	NR
Lin (2018) [37]	China	1	60	13/43	60.66 ± 6.07 E 60.83 ± 5.86 C	NR	NR	Chinese massage	Herbal application	WOMAC pain	-
Liu (2019) [38]	China	1	60	19/41	61.37 ± 10.31 E 62.83 ± 8.66 C	20.45 ± 7.27 E, M 21.63 ± 4.93 C, M	0–IV	Manual therapy	Herbal application	VAS	-
Peng (2018) [39]	China	1	98	44/54	55.19 ± 8.24 E 56.07 ± 9.24 C	1.82 ± 0.91 E, Y 1.78 ± 0.85 C, Y	NR	Chinese massage	Intra-articular injection	VAS and WOMAC pain	NR
Perlman (2006) [40]	USA	2	68	15/53	70.40 ± 11.30 E 66.20 ± 11.30 C	NR	NR	Swedish massage	Usual care	VAS	+
Perlman (2012) [41]	USA	2	50	38,12	62.60 ± 10.60 E 63.60 ± 10.20 C	NR	NR	Swedish massage	Usual care	VAS	-
Qu (2020) [42]	China	1	100	42/58	43–60	NR	NR	Chinese massage	Herbal application	VAS	NR
Wu (2015) [43]	China	1	60	18/42	63.37 ± 5.76 E 64.58 ± 4.24 C	5.26 ± 3.21 E, Y 4.86 ± 3.73 C, Y	NR	Manual therapy	Usual care	WOMAC pain	NR
Xu (2021) [44]	China	1	91	13/74	70.07 ± 3.85 E 69.80 ± 3.51 C	40.18 ± 19.56 E, M 39.81 ± 19.19 C, M	I-III	Chinese massage	Oral analgesics	VAS	+
Yao (2018) [45]	China	1	72	33/39	57.34 ± 11.56 E 56.82 ± 12.41 C	4.06 ± 1.25 E, Y 4.22 ± 1.47 C, Y	I-III	Chinese massage	Oral analgesics	VAS	NR
Yuan (2018) [46]	China	1	74	21/52	61.00 ± 9.70 E 58.20 ± 10.80 C	9.05 ± 7.90 E, Y 10.40 ± 8.50 C, Y	NR	Chinese massage	Oral analgesics	WOMAC pain	NR
Zhang (2014) [47]	China	1	120	95/25	61.82 ± 7.73 E 62.43 ± 6.13 C	8.29 ± 3.07 E, Y 8.81 ± 3.26 C, Y	NR	Chinese massage	Acupuncture	WOMAC pain	NR
Zhang (2018) [48]	China	1	60	41/19	59.80 ± 7.25 E 63.40 ± 4.98 C	9.20 ± 2.50 E, Y 8.80 ± 1.40 C, Y	NR	Chinese massage	Acupuncture	VAS	NR
Yang (2022) [49]	China	1	448	134/314	54.95 ± 11.26 E 58.26 ± 9.32 C	6.50 ± 4.76 E, Y 5.00 ± 6.12 C, Y	I–II	Chinese massage	Acupuncture	VAS	-
Xu (2022) [50]	China	1	68	31/37	58.00 ± 4.74 E 60.82 ± 7.40 C	18.62 ± 6.61 E, M 21.74 ± 10.12 C, M	I–III	Chinese massage	Cupping	VAS	-
Ma (2022) [51]	China	1	74	33/41	54.17 ± 8.62 E 54.78 ± 9.13 C	30.54 ± 42.17 E, M 31.24 ± 41.76 C, M	I–III	Chinese massage	Intra-articular injection	VAS	-
Liu (2022) [52]	China	1	90	29/61	58.80 ± 7.60 E 58.90 ± 7.80 E 58.80 ± 7.10 C	5.30 ± 2.50 E, Y 5.40 ± 2.90 E, Y 5.40 ± 3.30 C, Y	I–III	Chinese massage	Exercise therapy	VAS	-
Liu (2023) [53]	China	1	96	38/58	60.33 ± 3.44 E 59.56 ± 3.57 C	NR	0–III	Chinese massage	Usual care	VAS	+
Guo (2022) [54]	China	1	151	71/80	62.00 ± 7.80 E 61.50 ± 7.20 C 63.20 ± 8.00 C	5.80 ± 4.80 E, Y 6.20 ± 5.50 C, Y 5.60 ± 5.90 C, Y	NR	Chinese massage	Usual care/exercise therapy	VAS and WOMAC pain	NR

*E* experimental group, *C* control group, *K/L* Kellgren-Lawrence + with adverse events, -without adverse events, *NR* unreported, *Y* years, *M* months, *VAS* visual analogue scale, *WOMAC* Western Ontario and McMaster Universities Osteoarthritis Index

### VAS score

**Fig. 3:**
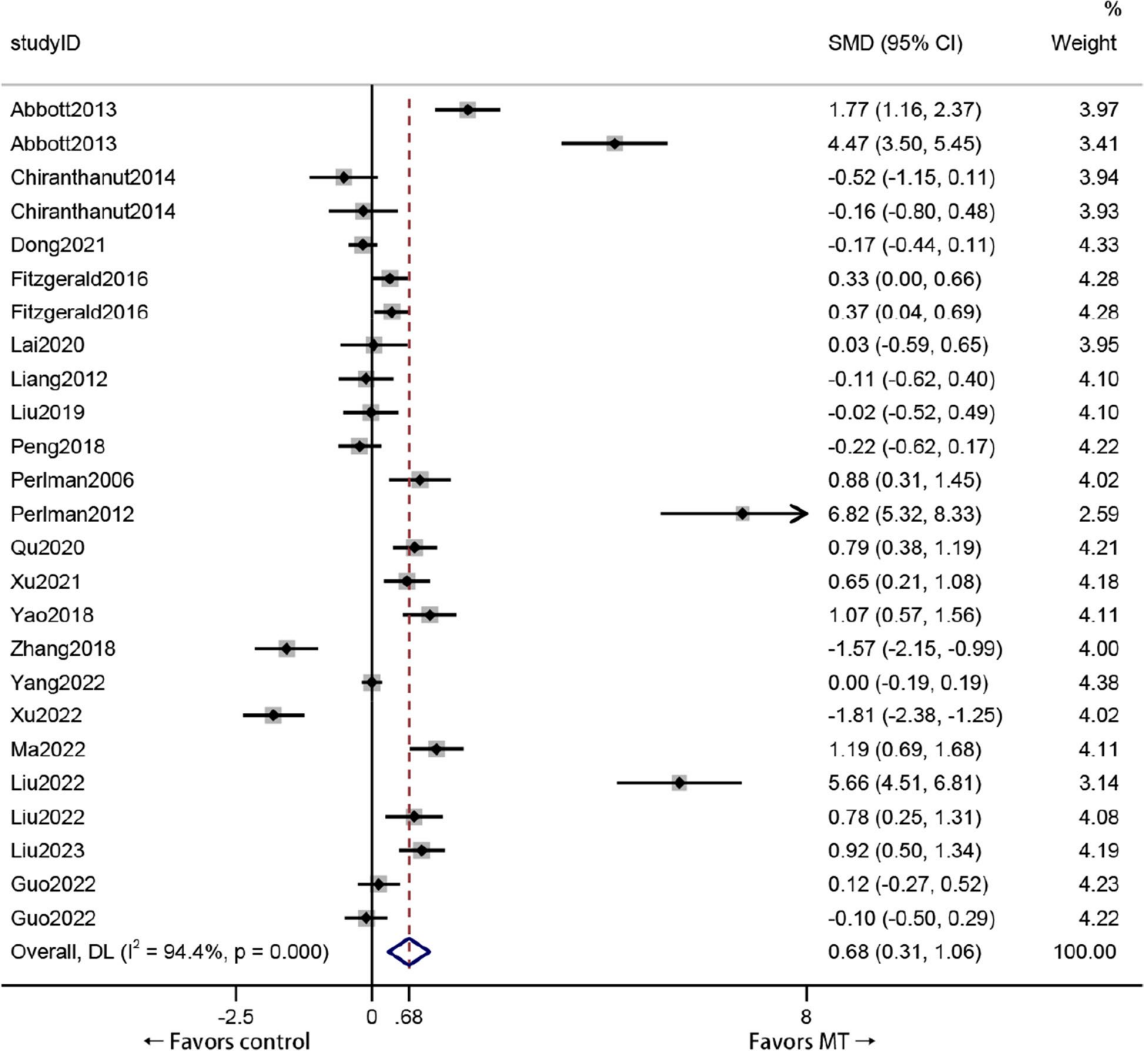
Forest plot comparing the VAS score reductions of KOA patients who received MT and other treatments

Patients treated with MT had a significantly greater VAS score than did those who received other therapies (standardized mean difference (SMD) 0.68, 95% *CI*: 0.31 to 1.06), with a significantly greater effect size (*Z* = 3.56, *P* < 0.01); however, the effect was considered small. Significant heterogeneity was found among the enrolled studies (*I* ^2^ = 94.4%, *P* = 0.000) (Fig. 3).

### Compared with other therapies

**Fig. 4:**
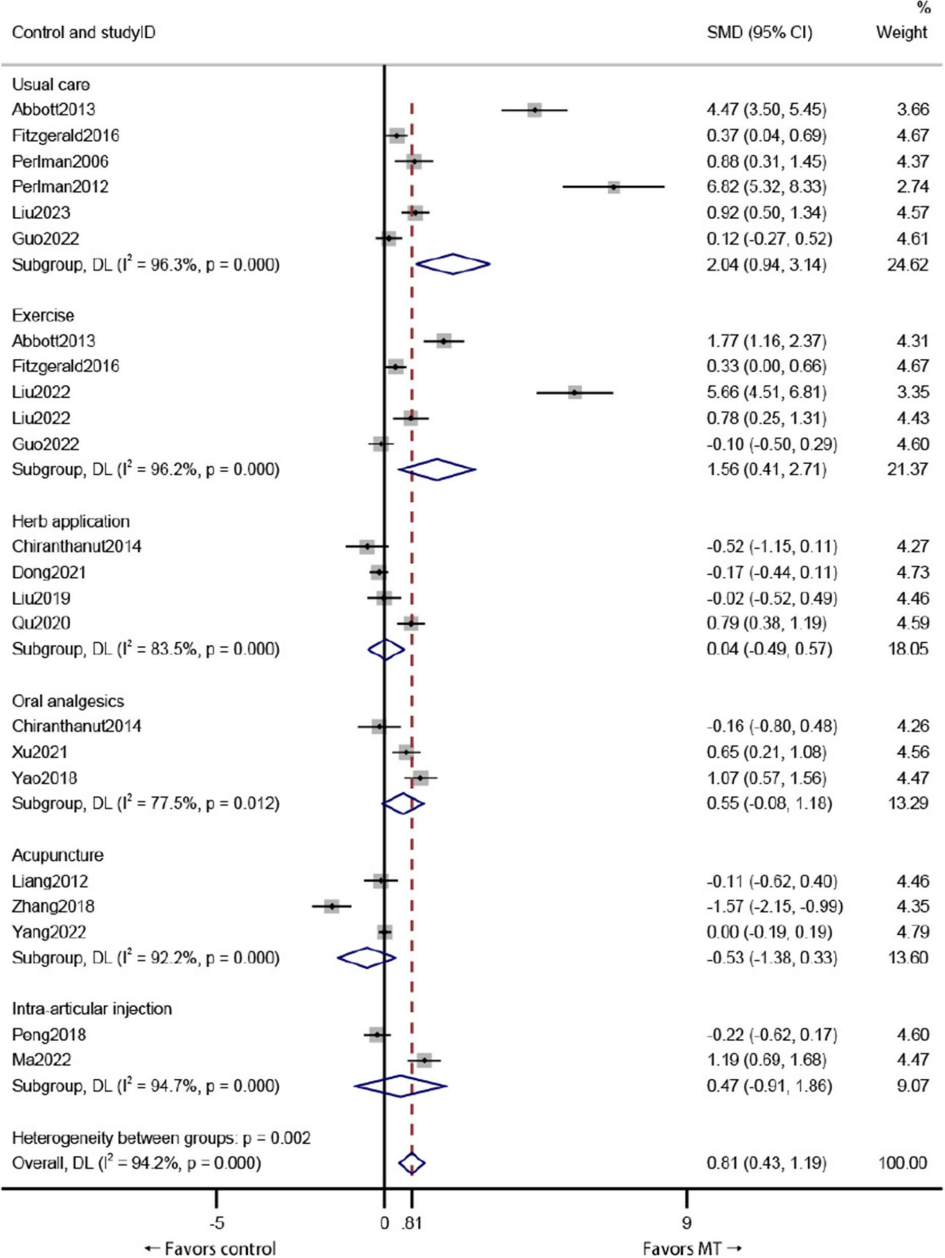
Subgroup analysis of VAS score outcomes based on the type of intervention in the control group

The results of subgroup analysis based on the type of intervention showed that compared with those in the usual care group, improvements in the VAS score were more significant in the MT group (*P* = 0.000). Moreover, improvements in the VAS score in the MT group were significant (*P* = 0.008). However, compared with that in the herbal application, oral analgesic, acupuncture, and intra-articular injection groups, the VAS score in the MT group showed no significant improvement (*P* > 0.05) at the end of intervention (Fig. 4).

### Course

**Fig. 5:**
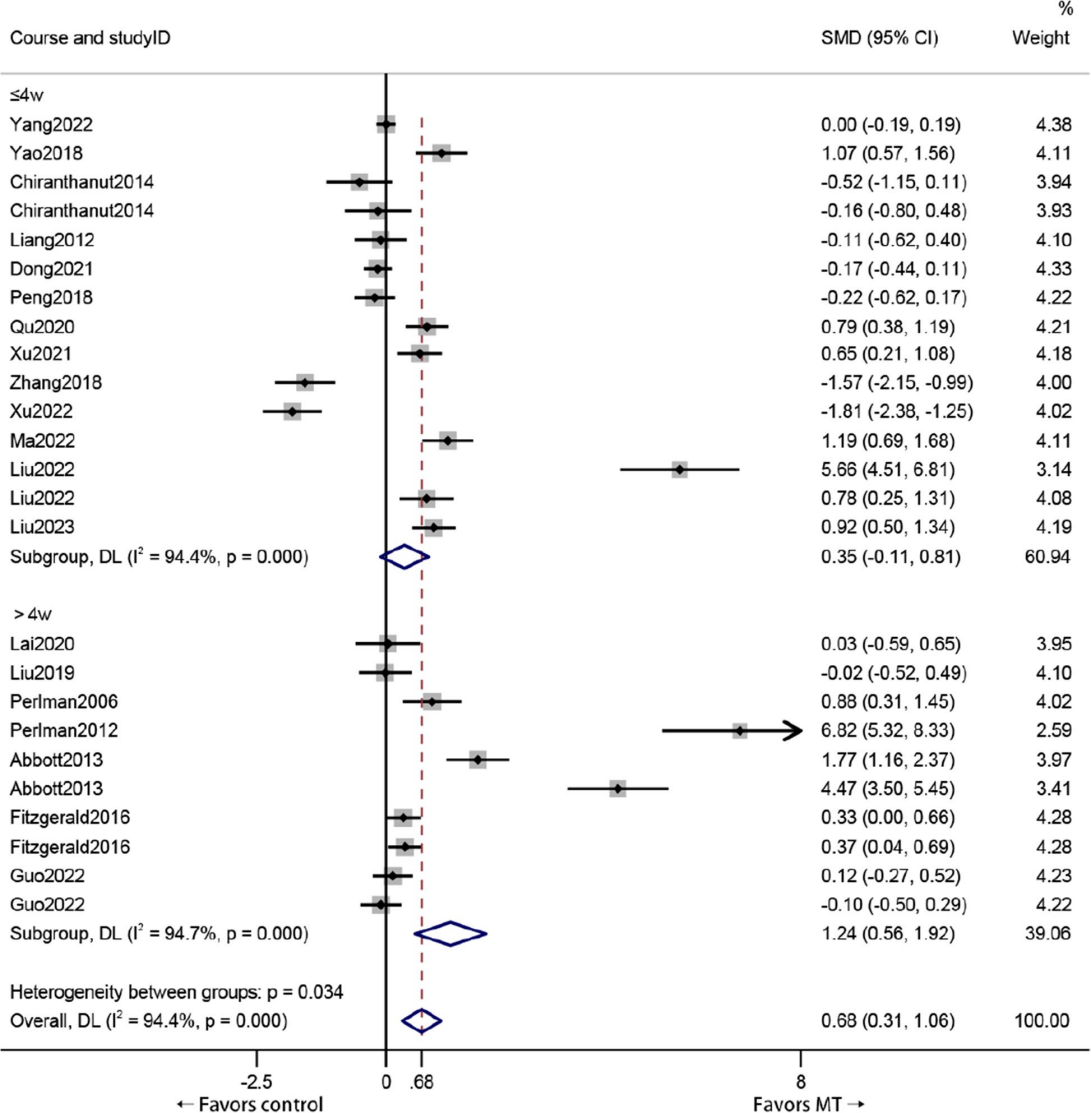
Subgroup analysis of VAS score outcomes based on course of treatment

Compared with other interventions, MT treatments of more than 4 weeks (*P* = 0.000) may be superior to treatments of less than or equal to 4 weeks (*P* = 0.136) in terms of improvement in VAS score (Fig. 5).

#### Secondary outcome

### The effect of MT compared with other therapies on the WOMAC pain score in KOA patients

**Fig. 6:**
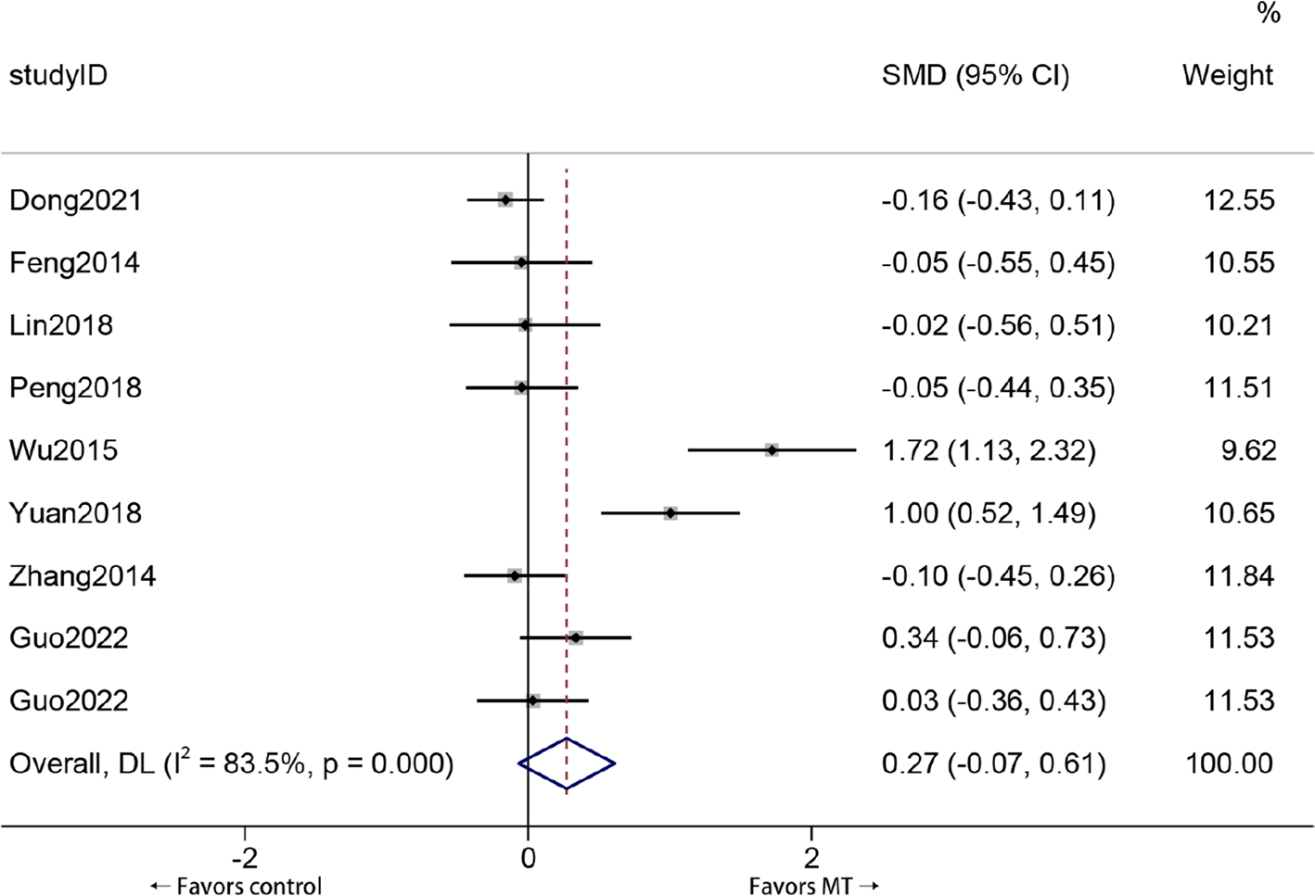
Forest plot comparing the WOMAC pain score reductions of KOA patients who received MT and other treatments

Eight trials (9 cohorts) that included 877 patients reported changes in the WOMAC pain scores of KOA patients who received MT or other therapies. As shown in Fig. 6, compared with patients who received other therapies, patients treated with MT did not show significant changes in WOMAC pain scores (standardized mean difference (SMD) 0.27, 95% *CI*: − 0.07 to 0.61), with a small effect size (*Z* = 1.561, *P* = 0.119). Significant heterogeneity was found among the studies (*I* ^2^ = 83.5%, *P* = 0.000). MT failed to improve the WOMAC pain score of KOA patients compared with that of patients receiving other therapies.

#### Follow-up and treatment cycle

The included studies ranged from 1.5 to 9 weeks of treatment for MT. Eight studies [30, 32, 34, 37, 39–41, 54] reported follow-up results, with a minimum follow-up of 1 month and a maximum follow-up of 1 year, as shown in Table 3. In terms of pain relief, 6 [30, 32, 37, 39–41] of these follow-ups found moderate to satisfactory long-term effects of MT in the treatment of KOA, 1 [34] study showed only short-term benefits for MT, and 1 [54] study did not report follow-up statistics.

**Table 3:** MT treatment cycle and follow-up in pain

Author, year	Follow-up			Treatment (week)
	Period	Effect	Description	
Abbott, 2013 [30]	1 year	+	The improvements in pain and disability were evident at 9 weeks and sustained for a year.	9
Chiranthanut, 2014 [31]	NR	NR	NR	3
Dong, 2021 [32]	3-6 month	+-	Mild recurrence of symptoms and signs during follow-up time (22/105). No severe relapses seen	4
Feng, 2014 [33]	NR	NR	NR	4
Fitzgerald, 2016 [34]	1 year	-	MT may have some short term benefit.	9
Lai, 2020 [35]	NR	NR	NR	6
Liang, 2012 [36]	NR	NR	NR	3
Lin, 2018 [37]	1, 3 month	+	The 1-month and 3-month follow-ups were showed a more consistent treatment effect than after 4 weeks of treatment.	4
Liu, 2019 [38]	NR	NR	NR	8
Peng, 2018 [39]	6 month	+	At the 6-month follow-up after MT treatment, VAS scores were significantly lower compared with pre-treatment	4
Perlman, 2006 [40]	16 week	+	MT was largely sustained at the 16-week follow-up visit.	8
Perlman, 2012 [41]	16, 24week	+	MT showed improvements.	8
Qu, 2020 [42]	NR	NR	NR	4
Wu, 2015 [43]	NR	NR	NR	8
Xu, 2021 [44]	NR	NR	NR	4
Yao, 2018 [45]	NR	NR	NR	2
Yuan, 2018 [46]	NR	NR	NR	3
Zhang, 2014 [47]	NR	NR	NR	4
Zhang, 2018 [48]	NR	NR	NR	4
Yang, 2022 [49]	NR	NR	NR	1.5
Xu, 2022 [50]	NR	NR	NR	4
Ma, 2022 [51]	NR	NR	NR	4
Liu, 2022 [52]	NR	NR	NR	4
Liu, 2023 [53]	NR	NR	NR	4
Guo, 2022 [54]	16 week	NR	NR	8

*MT* Manual therapy, *NR* Unreported; +: Satisfied; +-: Moderate; -: Not satisfied

#### Adverse reactions

Fourteen studies reported adverse events [30–32, 34, 37, 38, 40, 41, 44, 49–53]. Muscle soreness was the most common side effect of MT after treatment. Mild aggravation of pain occurred in one patient [44]. In most studies, adverse events were not reported.

#### Galbraith plot for analysis of heterogeneity across studies

**Fig. 7:**
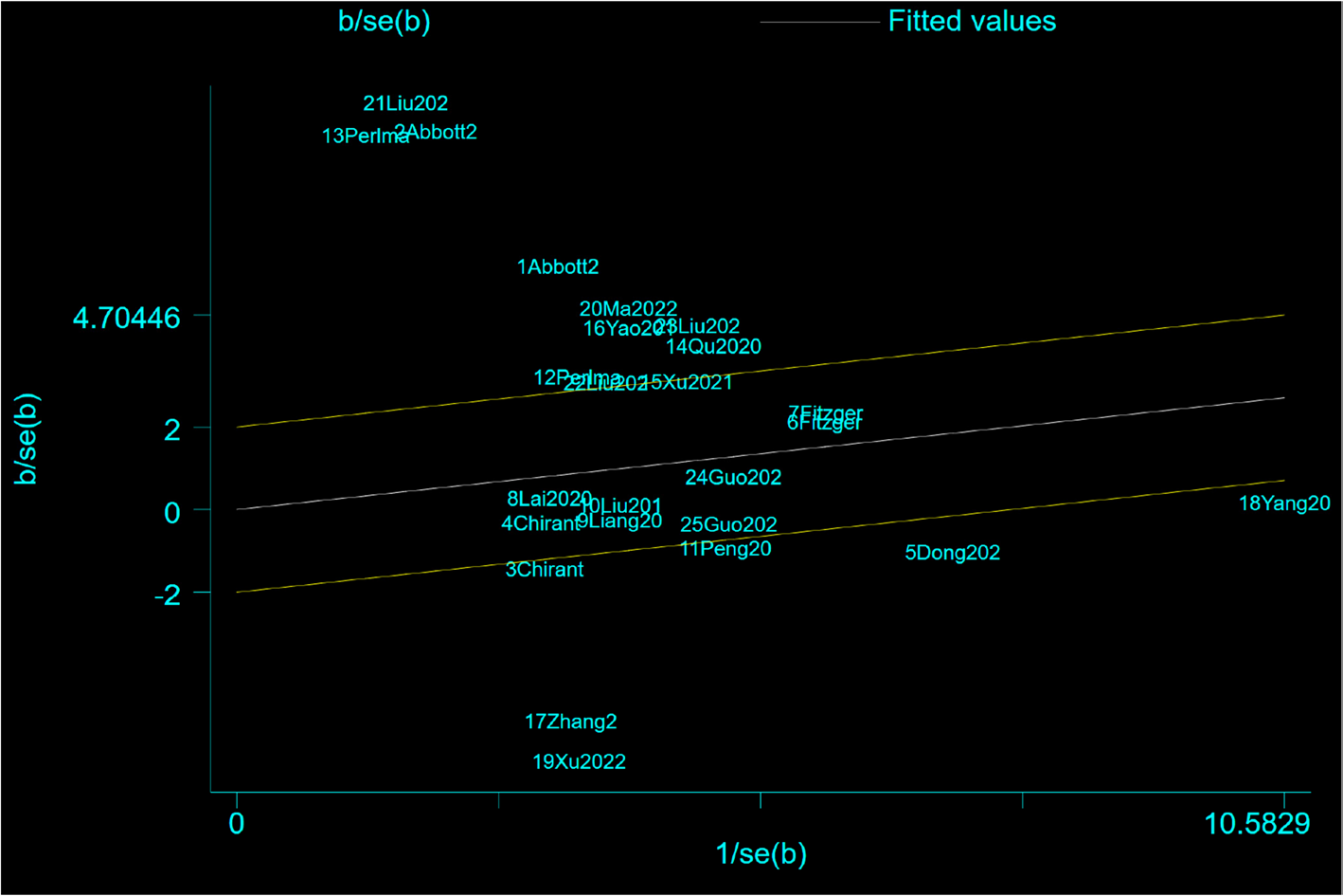
Using Galbraith plot to infer studies that might be the source of heterogeneity

**Fig. 8:**
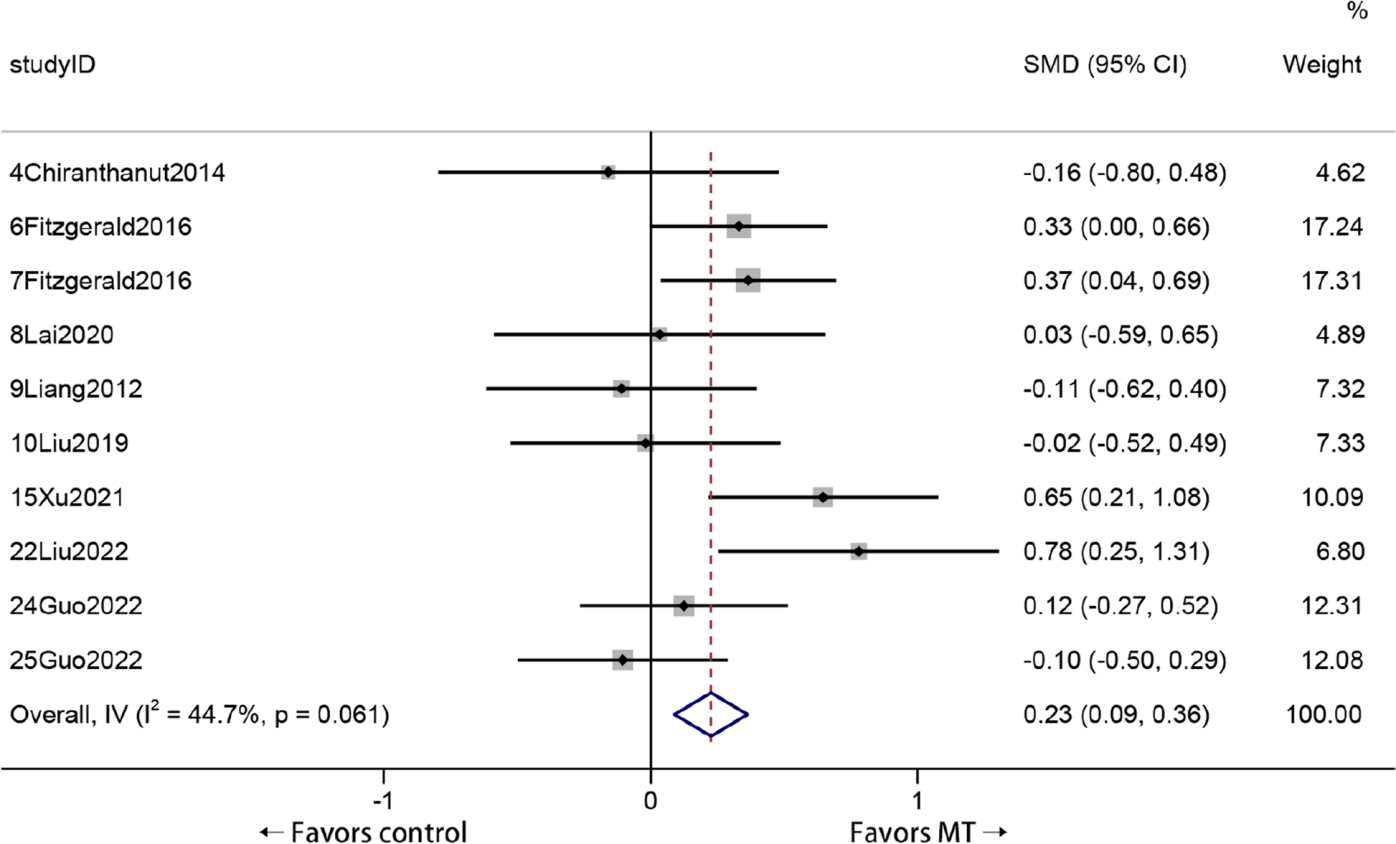
Comparing the VAS score reductions of KOA patients who received MT and other treatments with the remaining studies

Galbraith plots are an alternative to the forest plots proposed by Galbraith for visualizing the results of studies and meta-analyses, and Galbraith plots also aid in detecting sources of heterogeneity [55, 56]. Two lines are drawn at a vertical distance of ± 2 above and below the regression line, and these two lines together with the regression line constitute an interval. According to the relevant literature, studies that were published completely outside this interval may be responsible for the observed heterogeneity [33]. Therefore, we excluded all 15 studies outside the interval and performed a secondary analysis on the remaining 10 studies (Fig. 7). As shown in Fig. 8, the secondary analysis reached the same conclusion as before: compared with other treatments, MT was more effective at improving the VAS score of KOA patients (standardized mean difference (SMD) = 0.23, 95% *CI* = 0.009 to 0.36, *Z* = 3.233, *P* = 0.001). Moreover, the heterogeneity between studies was reduced (*I* ^2^ = 44.7%, *P* = 0.061).

#### Publication bias and sensitivity analysis

In this meta-analysis, we assessed publication bias using funnel plots and Egger’s test [57], and our results indicated no significant publication bias (*P*_egger_ = 0.442) in the included studies (Appendix 4). We examined the effect of classifying MT with other therapies in sensitivity analysis, which did not affect our results. Sensitivity analysis indicated that the results were stable (Appendix 5).

### Meta-regression

As differences in the treatment period, patient age, and sex ratio may have affected the analysis results, we performed meta-regression to evaluate whether the above factors had a significant impact on the results. Overall, the treatment duration, mean age of patients, and sex ratio did not significantly affect the results (Appendix 6).

## Discussion

This study constitutes an update of a comprehensive systematic review to assess the efficacy of MT in conservative management of patients with KOA for pain relief, with more stringent inclusion criteria to ensure the quality of the source RCTs. Considerable effort was made to conduct an extensive literature search. Twenty of the 25 studies were conducted in China, 3 in the USA, and 1 each in New Zealand and Thailand. Most of the included studies used MT.

This study integrated evidence from existing clinical RCTs of MT for KOA pain and identified proposed research targets that remain to be addressed. Our findings on VAS score improvement in patients with KOA were statistically significant but may not be clinically meaningful when compared to those for patients receiving other therapies in addition to usual care and exercise. Although no significant differences in WOMAC pain scores were found for the secondary outcomes, MT appears to have the potential and is undeniably effective at reducing pain in KOA patients. In this meta-analysis, we used the MCID as a threshold to assess the clinical significance of the difference between the MT group and the control group rather than relying solely on statistical significance. There are anchor-based and distribution-based methods for calculating the MCID, and based on previous studies, we set the threshold at 20% using the anchor-based method [58–60]. According to our subgroup analyses, the effect of MT on the VAS score at the end of the intervention exceeded the effect of the MCID in both the usual care and exercise groups but not in other control groups. These findings suggest that MT may have better clinical applicability in the short term than usual care and exercise. The findings, including the Chinese database search, support previous meta-analyses in the literature [61]. Although guidelines [62] report that exercise treatment might be more effective than MT at reducing pain intensity at short-term follow-up, our meta-analysis showed that MT was no less effective than exercise interventions for pain relief in patients with KOA and was superior to usual care. Nevertheless, because most of the studies included in this systematic review were carried out in China, the results may be influenced to some extent by the limitations of factors such as geographic location and cultural group, and additional dimensions of data should be added in the future to assess the stability of the findings.

In terms of outcome indicators, MT did not significantly differ with regard to WOMAC pain score, indicating that MT has limitations in relieving pain and does not cover the full range of patients’ daily lives, especially for those in pain situations such as walking, moving up and downstairs, sleeping, standing, sitting, and lying down. However, the VAS for global pain had a slightly greater assay sensitivity at the trial and meta-analysis levels than the WOMAC pain subscale [63]. In contrast to comprehensive assessment of the efficacy of the VAS, the WOMAC pain score consists of five questions, and the results are inherently multidimensional; e.g., the pain relief effect of MT might be limited in some specific activities, resulting in biased scores, but this needs to be validated by additional studies [64]. Furthermore, RCTs of MT for KOA treatment are still rare because of the lack of research on long-term treatment in particular, and additional RCTs on the effects of MT for KOA treatment are needed, as are studies integrating MT with biomechanics.

MT appears to be safe for individuals with KOA. In our review, 56% of the included studies reported safety information, with few adverse events described and no serious adverse events shown. A meta-analysis of adverse events of MT in RCTs found that most of the observed adverse events were musculoskeletal related, transient in nature, or mild to moderate in severity [65]. Our review also supports this conclusion by revealing that muscle discomfort and pain caused by MT were the most common adverse events. However, most existing meta-analyses on MT in KOA patients do not include safety/adverse effects because poor reporting of safety information in MT clinical trials is common in other populations. We strongly suggest that future investigators monitor and report safety/adverse events of MT in clinical trials for KOA, as recommended by CONSORT.

We could better explain the effects if more attention was given to the qualitative components of the intervention, such as the context of the visit, patient beliefs, and preferences. Although we focused on the impact of MT on the level of pain in KOA patients in this review, the economic costs associated with care and psychophysiological mechanisms should also be considered. In particular, this approach has the following advantages: changes in parasympathetic activity (as measured by heart rate, blood pressure, and heart rate variability) and hormonal levels (as measured by cortisol levels) following MT result in a relaxation response (physiological mechanisms); a reduction in anxiety and an improvement in mood state after MT cause relaxation (psychological mechanisms) [66]. The treatment cycle of MT is typically 1.5 to 9 weeks, and follow-up results show that MT is more effective for pain relief in the short term and appears to have improved efficacy in the long term, which may be maintained for up to 1 year; however, the outcome may not be completely stable or reliable. One study [34] indicated that MT results were unsatisfactory at follow-up to 1 year. This may also be limited by the short treatment period of MT, and the effectiveness and long-term efficacy of interventions with long MT sessions may be worth exploring. Furthermore, the most recent systematic review [67] on the cost-effectiveness of noninvasive and nonpharmacologic interventions for KOA concluded that studies of other economic assessments of MT for KOA are rare. For example, with other conservative treatment options, assessing whether a long course of MT treatment (> 4 weeks) provides the best economic benefit, and therefore, whether MT is a cost-effective option for treating KOA remains to be determined.

### Limitations

First, because of the different settings and populations (age, occupation, and socioeconomic status) in the studies and use of different recruitment methods and MT techniques, we could not perfectly address the issues related to statistical heterogeneity. Although we implemented subgroup analyses of different techniques (results not shown) and validated the results using Galbraith plots, such results will be difficult to address in future reviews. Second, some studies were excluded, even though WOMAC pain scores were collected. These studies did not report methods for evaluating WOMAC pain or matching our inclusion criteria, which made it difficult to integrate the WOMAC pain data. Finally, most studies ignore classifying the severity of KOA unreported in the Kellgren–Lawrence grading system. Hence, the inconsistency of the severity of the disease limits the number of studies, and we were unable to conduct subgroup analysis according to the severity of the disease to explore its effect in more depth.

### Implications

The findings suggest that MT as a stand-alone treatment may not produce satisfactory meaningful pain relief, especially in some elderly KOA patients who present with pain only in certain circumstances. The method of adverse event assessment was unclear. In general, MT may result in a temporary mild increase in muscle soreness but is essentially safe. Therefore, if a patient chooses nonsurgical conservative treatment, clinicians treating KOA patients may safely prioritize MT in anticipation of a possible short-term improvement in pain.

## Conclusions

MT is potentially effective at reducing pain in KOA patients, and long-term treatment periods may be more effective. While there may be limitations to the effectiveness of MT, it may be more advantageous than usual care and exercise therapy, but the results need to be referenced with caution. MT seems to be safe in patients with KOA, but better monitoring and reporting of security information are strongly recommended. Overall, there is a lack of large samples of randomized controlled trials and active research evaluating the economic benefits of MT. More high-quality studies are needed in the future to determine the beneficial effects of MT on pain in patients with KOA.

### Supplementary Information

**Additional file 1:** **Appendix 1.** Search terms and strategies. **Appendix 2.** Detailed risk of bias judgment by domains for Stress (Rob2 Tool): at the end of the intervention. **Appendix 3.** The GRADE approach to evidence synthesis and operationalization of criteria items. **Appendix 4.** Funnel plot for VAS score. **Appendix 5.** Sensitivity analysis for VAS score. **Appendix 6.** Meta-regression (Table S1–5).

## Data Availability

The datasets supporting the conclusions of this article are included within the article.
