# Supplementary material for: The effects of manual therapy in pain and safety of patients with knee osteoarthritis: a systematic review and meta-analysis
Source: Syst Rev. 2024 Mar 19;13:91. doi: 10.1186/s13643-024-02467-7 (PMC10949788; doi:10.1186/s13643-024-02467-7)
Supplement: Supplementary file 1 — Additional file 1: Appendix 1. Search terms and strategies. Appendix 2. Detailed risk of bias judgment by domains for Stress (Rob2 Tool): at the end of the intervention. Appendix 3. The GRADE approach to evidence synthesis and operationalization of criteria items. Appendix 4. Funnel plot for VAS score. Appendix 5. Sensitivity analysis for VAS score. Appendix 6. Meta-regression (Table S1–5). [file 13643_2024_2467_MOESM1_ESM.docx]

**Appendices**

**Appendix 1 Search terms and strategies**

**Appendix 2 Detailed risk of bias judgment by domains for Stress (Rob2 Tool): at the end of the intervention**

**Appendix 3 The GRADE approach to evidence synthesis and operationalization of criteria items**

**Appendix 4 Funnel plot for VAS score**

**Appendix 5 Sensitivity analysis for VAS score**

**Appendix 6 Meta-regression (Table S1-5)**

**Appendix 1: Search terms and strategies**

| Databases | Search strategies |
| --- | --- |
| CNKI | SU=“按摩”+“按摩疗法”+“推拿治疗”+“推拿疗法”+“推拿”+ 手法 AND SU=“膝关节骨性关节炎”+“膝骨关节炎”+“膝骨性关节炎”+“膝关节退行性关节炎”+“膝关节骨关节炎”+ “膝痹” |
| Wanfang | （题名:(膝关节骨性关节炎 or 膝骨关节炎 or 膝骨性关节炎 or 膝关节退行性关节炎 or 膝关节骨关节炎 or 膝痹) or 摘要:(膝关节骨性关节炎 or 膝骨关节炎 or 膝骨性关节炎 or 膝关节退行性关节炎 or 膝关节骨关节炎 or 膝痹) ） and （题名:(推拿 or 按摩 or 手法 or 按摩疗法 or 推拿治疗 or 推拿疗法) or 摘要:(推拿 or 按摩 or 手法 or 按摩疗法 or 推拿治疗 or 推拿疗法) ） |
| China Science and Technology Journal Database (VIP) | （T=(膝关节骨性关节炎+膝骨关节炎+膝骨性关节炎+膝关节退行性关节炎+膝关节骨关节炎+膝痹) OR R=(膝关节骨性关节炎+膝骨关节炎+膝骨性关节炎+膝关节退行性关节炎+膝关节骨关节炎+膝痹) ） AND （T=（按摩+按摩疗法+推拿治疗+推拿疗法+推拿+手法) OR R=(按摩+按摩疗法+推拿治疗+推拿疗法+推拿+手法) ） |
| PubMed | #1 "Knee Joint"[Mesh] OR "Knee"[Mesh]  #2 Knee*[Title/Abstract]  #3 1788/01/01: 2023/06/30[Date - Publication]  #4 (#1 OR #2) AND #3  #5 "Arthritis"[Mesh] OR "Osteoarthritis"[Mesh] OR "Osteoarthritis, Knee"[Mesh]  #6 Arthrit*[Title/Abstract] OR Osteoarthr*[Title/Abstract]  #7 1801/01/01: 2023/06/30[Date - Publication]  #8 (#5 OR #6) AND #7  #9 "Massage"[Mesh] OR "Musculoskeletal Manipulations"[Mesh]  #10 massage*[Title/Abstract] OR "Zone Therap*"[Title/Abstract] OR Manipul*[Title/Abstract]  #11 1857/01/01: 2023/06/30[Date - Publication]  #12 (#9 OR #10) AND #11  #13 WOMAC[Text Word]  #14 "Visual Analog Scale"[Mesh] OR "Visual Analog Scale"[Text Word] OR "VAS"[Text Word]  #15 #13 OR #14  "Randomized Controlled Trial"[Publication Type] OR "Randomized Controlled Trials as Topic"[MeSH Terms] OR "Controlled Clinical Trial"[Publication Type] OR "Random Allocation"[MeSH Terms] OR "Double-Blind Method"[MeSH Terms] OR "Single-Blind Method"[MeSH Terms] OR "clinical trial, phase ii"[Publication Type] OR "clinical trial, phase iii"[Publication Type] OR "clinical trial, phase iv"[Publication Type] OR "Controlled Clinical Trial"[Publication Type] OR "Randomized Controlled Trial"[Publication Type] OR ("clinic*"[Title/Abstract] AND "trial*"[Title/Abstract] AND ("randomized"[Title/Abstract] OR "placebo"[Title/Abstract] OR "randomly"[Title/Abstract] OR "blind*"[Title/Abstract] OR "mask*"[Title/Abstract])) |
| Cochrane Library | #1 MeSH descriptor: [Knee] explode all trees  #2 (knee*):ti,ab,kw  #3 #1 OR #2  #4 MeSH descriptor: [Arthritis] explode all trees OR MeSH descriptor: [Osteoarthritis] explode all trees  #5 (arthrit* OR osteoarthr*):ti,ab,kw  #6 #4 OR #5  #7 MeSH descriptor: [Massage] explode all trees OR MeSH descriptor: [Massage] explode all trees  #8 massage*:ti,ab,kw OR 'zone therap*':ti,ab,kw OR manipul*:ti,ab,kw  #9 #7 OR #8  #10 MeSH descriptor: [Visual Analog Scale] explode all trees  #11 ("western ontario and mcmaster universities osteoarthritis index*" OR womac OR "visual analog scale" OR VAS):ti,ab,kw  #12 #10 OR #11  #13 MeSH descriptor: [Randomized Controlled Trial] explode all trees OR MeSH descriptor: [Randomized Controlled Trials as Topic] explode all trees OR MeSH descriptor: [Controlled Clinical Trial] explode all trees OR MeSH descriptor: [Random Allocation] explode all trees OR MeSH descriptor: [Double-Blind Method] explode all trees OR MeSH descriptor: [Single-Blind Method] explode all trees OR (controlled clinical trial):pt OR (randomized controlled trial):pt  #14 (clinic* AND trial*):ti,ab,kw  #15 (random* OR placebo* OR blind* OR mask*):ti,ab,kw  #16 #17 OR (#18 AND #19)  #3 AND #6 AND #9 AND #12 AND #16 |
| Web of science | #1 AB=(knee*)  #2 AB=(arthrit* OR osteoarthr*)  #3 AB=(massage* OR "zone therap*" OR manipul*)  #4 "western ontario and mcmaster universities osteoarthritis index*" OR womac OR "visual analog scale" OR VAS  #5 ALL=("Randomized Controlled Trial" [Publication Type] OR "Randomized Controlled Trials as Topic"[Mesh] OR "Controlled Clinical Trial" [Publication Type] OR "Random Allocation"[Mesh] OR "Double-Blind Method"[Mesh] OR "Single-Blind Method"[Mesh] OR Clinical trial, phase II[Publication Type] OR Clinical trial, phase III[Publication Type] OR Clinical trial, phase IV[Publication Type] OR controlled clinical trial[Publication Type] OR randomized controlled trial[Publication Type])  #6 ((AB=(clinic* AND trial*)) AND AB=(randomized OR placebo OR randomly OR blind* OR mask*))  #7 #5 OR #6  #1 AND #2 AND #3 AND #4 AND #7 AND 2023 or 2022 or 2020 or 2019 or 2018 or 2016 or 2015 or 2020 or 2019 or 2018 or 2016 or 2015 or 2014 or 2013 or 2010 (出版年) |

**Appendix 2: Risk of bias judgment by domains for Pain (Rob2 Tool): at the end of the intervention**

| Total number of study = 25 | Randomization process | Deviations from intended interventions | Missing outcome data | Measurement of the outcome | Selection of the reported result | Overall Bias |
| --- | --- | --- | --- | --- | --- | --- |
| Low risk | 8 | 56 | 80 | 36 | 88 | 0 |
| Some concerns | 12 | 36 | 12 | 16 | 0 | 12 |
| High risk | 80 | 8 | 8 | 48 | 12 | 88 |

**Appendix 3: The GRADE approach to evidence synthesis and operationalization of criteria items**

| Comparisons | Risk of bias | Inconsistency | Indirectness | Imprecision | Publication bias | Quality of evidence |
| --- | --- | --- | --- | --- | --- | --- |
| MT vs usual care | Serious^a^ | Serious^b^ | Not serious | Not serious | Not serious | Low |
| MT vs exercise | Serious^a^ | Serious^b^ | Not serious | Not serious | Not serious | Low |
| MT vs herb application | Serious^a^ | Serious^b^ | Not serious | Not serious | Not serious | Low |
| MT vs Oral analgesics | Serious^a^ | Serious^b^ | Not serious | Serious^c^ | Not serious | Very low |
| MT vs Acupuncture | Serious^a^ | Serious^b^ | Not serious | Not serious | Not serious | Low |
| MT vs Intra-articular injection | Serious^a^ | Serious^b^ | Not serious | Serious^c^ | Not serious | Very low |
| MT vs Moxibustion | Serious^a^ | Serious^b^ | Not serious | Serious^c^ | Not serious | Very low |
| MT vs Cupping | Serious^a^ | Serious^b^ | Not serious | Serious^c^ | Not serious | Very low |

a: Selection bias (random sequence generation, allocation hiding, baseline group similarity; b: When the heterogeneity or variability of results is large (e.g., I^2^ statistic values >50%, representing potentially substantial heterogeneity); c: Only one study or when there are multiple studies, the total number of events is less than 300.

**Appendix 4 Funnel plot for VAS score**


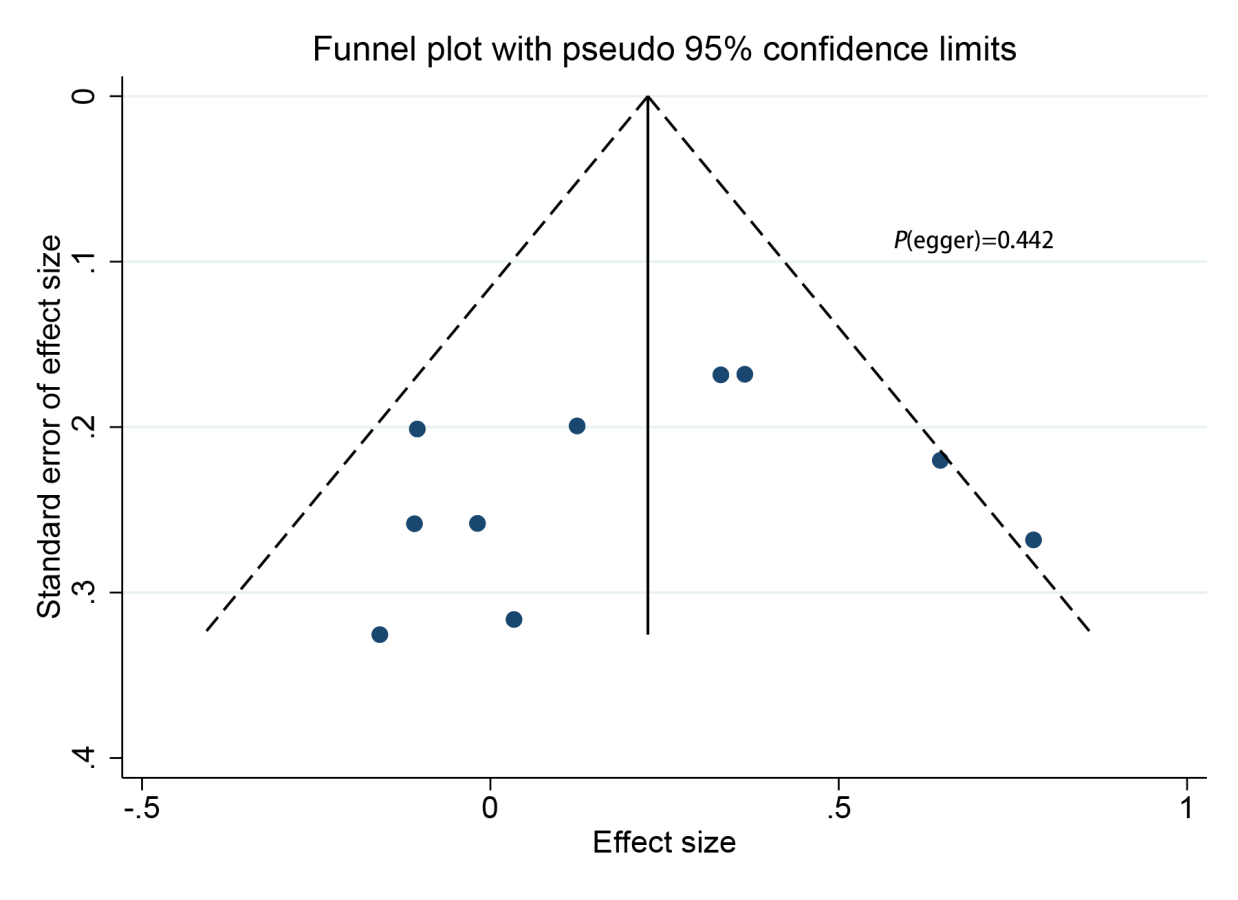


**Appendix 5 Sensitivity analysis for VAS score**


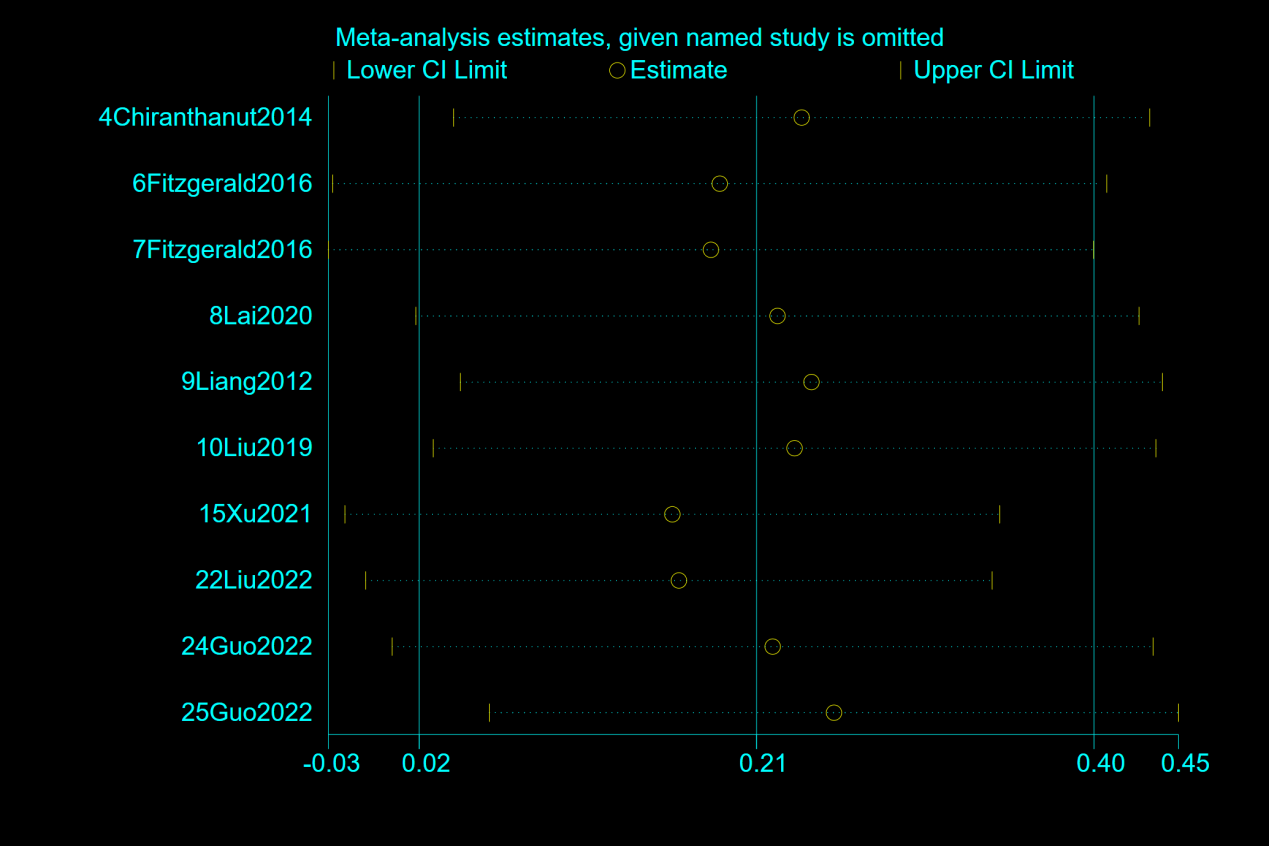


**Appendix 6 Meta-regression (Table S1-5)**

Table S1. Meta-regression analysis of the influence of treatment cycle on clinical outcomes

| _ES | exp(b) | Std. Err. | t | P>\|t\| | [95% Conf. Interval] | |
| --- | --- | --- | --- | --- | --- | --- |
| treatment cycle | -.0341805 | 0. 1610554 | -0.21 | 0.834 | -0.3673491 | 0.298988 |
| _cons | 0. 9796603 | 0. 953969 | 1.03 | 0.315 | -0.993775 | 2.953096 |

Table S2. Meta-regression analysis of the influence of average age of patients receiving massage therapy on clinical outcomes

| _ES | exp(b) | Std. Err. | t | P>\|t\| | [95% Conf. Interval] | |
| --- | --- | --- | --- | --- | --- | --- |
| average age (MT) | 0. 0138277 | 0. 0937356 | 0.15 | 0.884 | -0.1805679 | 0. 2082234 |
| _cons | -0.049591 | 5.763987 | --0.01 | 0.993 | -12.00337 | 11.90419 |

Table S3. Meta-regression analysis of the influence of average age of patients receiving other therapies on clinical outcomes

| _ES | exp(b) | Std. Err. | t | P>\|t\| | [95% Conf. Interval] | |
| --- | --- | --- | --- | --- | --- | --- |
| average age (other therapy) | -.0261386 | 0. 1063489 | -0.25 | 0.808 | -0.2466927 | 0.1944155 |
| _cons | 2.405017 | 6.549449 | 0.37 | 0.717 | -11.17771 | 15.98774 |

Table S4. Meta-regression analysis of the influence of proportion of female patients among patients receiving massage therapy on clinical outcomes

| _ES | exp(b) | Std. Err. | t | P>\|t\| | [95% Conf. Interval] | |
| --- | --- | --- | --- | --- | --- | --- |
| Female patients % (massage therapy) | -0.0253141 | 0.0236459 | -1.07 | 0.296 | -0.0743526 | 0.0237245 |
| _cons | 2.196281 | 1.368563 | 1.60 | 0.123 | -0.6419447 | 5.034507 |

Table S5. Meta-regression analysis of the influence of proportion of female patients among patients receiving other therapies on clinical outcomes

| _ES | exp(b) | Std. Err. | t | P>\|t\| | [95% Conf. Interval] | |
| --- | --- | --- | --- | --- | --- | --- |
| Female patients % (other therapies) | -0.0185203 | 0.0225413 | -0.82 | 0.420 | -0.0652681 | 0.0282275 |
| _cons | 1.788547 | 1.273497 | 1.40 | 0.174 | -0.8525243 | 4.429619 |
